# Flavor Profiling Using Comprehensive Mass Spectrometry Analysis of Metabolites in Tomato Soups

**DOI:** 10.3390/metabo12121194

**Published:** 2022-11-29

**Authors:** Simon Leygeber, Justus L. Grossmann, Carmen Diez-Simon, Naama Karu, Anne-Charlotte Dubbelman, Amy C. Harms, Johan A. Westerhuis, Doris M. Jacobs, Peter W. Lindenburg, Margriet M. W. B. Hendriks, Brenda C. H. Ammerlaan, Marco A. van den Berg, Rudi van Doorn, Roland Mumm, Robert D. Hall, Age K. Smilde, Thomas Hankemeier

**Affiliations:** 1 Leiden Academic Centre for Drug Research, Leiden University, Einsteinweg 55, 2333 CC Leiden, The Netherlands; 2 Swammerdam Institute for Life Sciences, University of Amsterdam, Science Park 904, 1098 XH Amsterdam, The Netherlands; 3 Laboratory of Plant Physiology, Wageningen University and Research, Droevendaalsesteeg 1, 6708 PB Wageningen, The Netherlands; 4 Unilever’s Foods Innovation Centre, Bronland 14, 6708 WH Wageningen, The Netherlands; 5 Leiden Centre for Applied Bioscience, University of Applied Sciences Leiden, Zernikedreef 11, 2333 CK Leiden, The Netherlands; 6 DSM Center for Biodata & Translation, Alexander Fleminglaan 1, 2613 AX Delft, The Netherlands; 7 DSM Food & Beverages, Alexander Fleminglaan 1, 2613 AX Delft, The Netherlands; 8 Wageningen Research (Bioscience), Wageningen University and Research, Droevendaalsesteeg 1, 6708 PB Wageningen, The Netherlands

**Keywords:** metabolomics, chemometrics, food, GC-MS, LC-MS, sensory evaluation, tomato soup, yeast

## Abstract

Trained sensory panels are regularly used to rate food products but do not allow for data-driven approaches to steer food product development. This study evaluated the potential of a molecular-based strategy by analyzing 27 tomato soups that were enhanced with yeast-derived flavor products using a sensory panel as well as LC-MS and GC-MS profiling. These data sets were used to build prediction models for 26 different sensory attributes using partial least squares analysis. We found driving separation factors between the tomato soups and metabolites predicting different flavors. Many metabolites were putatively identified as dipeptides and sulfur-containing modified amino acids, which are scientifically described as related to umami or having “garlic-like” and “onion-like” attributes. Proposed identities of high-impact sensory markers (methionyl-proline and asparagine-leucine) were verified using MS/MS. The overall results highlighted the strength of combining sensory data and metabolomics platforms to find new information related to flavor perception in a complex food matrix.

## 1. Introduction

The quality of a food product is determined by multiple sensory properties, such as taste, odor, color and texture. Food innovation aims to create novel sensations or enhance specific sensory properties that are preferred by the consumer. The food industry also seeks to diminish undesirable sensory properties, for example, the appearance of off-flavors during product processing and storage prior to consumption. These innovations in sensory quality can be achieved by altering the product composition in a targeted manner, possibly at the molecular level.

Traditionally, the approach used to assess flavor perception is through trained sensory panels alone, grading sensory attributes within the categories of odor, flavor, mouthfeel, aftertaste and afterfeel. The sensomics approach gives an opportunity to link molecular information to specific sensory attributes. Applying sensomics can reduce the number of samples that need to be evaluated using senses during product development. In addition, to ensure objectiveness, significance and reproducibility, repetition of the sensory testing by multiple assessors is necessary. Overall, this makes sensory panels both costly and time-consuming. The interactions of specific flavors or odors within complex matrices are vastly unknown. Due to this limited insight, food development is still largely based on trial-and-error approaches. If sensory attributes could be directly related to (combinations of) chemical species, then measuring these could substitute or minimize sensory panel sessions during the development of new ingredients and food products [1]. This would help to establish a data-driven approach to determine the effect of food matrices and chemical profiles for sensory attributes [2,3,4]. 

Metabolomics approaches allow for the analysis of a wide range of small molecules from different chemical classes and have been used widely, especially in the search for biomarkers of diseases and a general state of health. However, metabolomics has also been increasingly applied to many other areas, including food production and development. Examples of this are the measurement of contaminants or biomarkers for impure or toxic food batches in industrial food production or storage [5,6,7]. In addition, food metabolomics has proven useful for determining differences in the chemical composition of food products and beverages related to the origin of the ingredients and raw materials or in the production process, such as fermentation or heating [8,9,10,11]. While the combination of sensory analysis and metabolomics has thus far only been applied to some products [2,11,12,13,14], the development of new food sensations will strongly benefit from such a combined approach.

Tomato soup is a popular food in many cultures with many varieties, including added spices and flavors. Yeast extracts and process flavors are usually added to enhance the flavor intensity or the spiciness or salty sensations. The detailed sensorial impact of yeast products in a complex food matrix is not known. Thus, we designed a study where the dosages of different yeast products and the tomato soup itself were systematically varied. In addition, the tomato soups were composed of different oil types and simmered for either a short or long time to find out whether heat treatment and the interaction with the oil can alter the sensory perception. These deliberate and systematic changes in the tomato soup formulation using a simplified recipe also allowed us to study the link between molecular composition and sensory properties.

Flavor perception is unique for individual consumers and is based on complex interactions of food components with human taste and smell receptors. It is important for this study to distinguish between consumer panels, which are focused on the preferences of taste, and trained sensory panels, which quantify sensory attributes based on a qualitative data analysis (QDA) without personal preferences. The latter was used in this study to allow us to investigate sensory data without the influence of individual preferences.

The aim of this study was to evaluate the use of metabolomics to predict sensory profiles of tomato soups that had differences in yeast-derived products and dosage, tomato content, oils and heating. We applied gas chromatography–mass spectrometry (GC-MS) and liquid chromatography–mass spectrometry (LC-MS) for the volatile and non-volatile chemical profiling of the soups to provide complementary information and then linked this to sensory panel data. We used this approach for a data-driven strategy to select features that relate to sensory attributes and compositional properties. To illustrate this concept, we identified some of the highest-impact features to gain new insights for the prediction of flavor.

## 2. Materials and Methods

**Figure 1:**
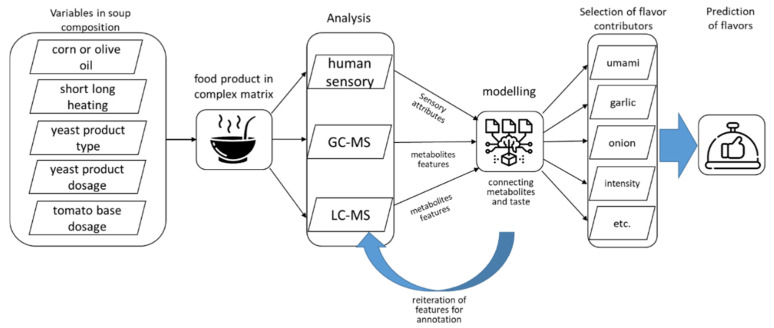
Schematic overview of the approach, which addressed the influence of soup composition and preparation by modeling the relation between sensory attributes and their main molecular contributors (characterized by three different analytical platforms) (icons from Flaticon.com, accessed on 1 June 2021).

The overview of the sample composition and the combination of metabolomics and sensory platforms to predict flavor qualities is illustrated in Figure 1.

### 2.1. Soup Composition

A total of 27 contrasting tomato soup samples were used as described in Davarzani et al. (2021) [15]. The ingredients were base mixes of tomato powder, sucrose, roux, starch, oil, salt, lemon juice, pepper (Unilever R&D, Wageningen, The Netherlands) and yeast-derived flavor products (DSM, Delft, The Netherlands). Five compositional factors—oil type (corn, olive), tomato dosage (high, low), yeast product, yeast dosage (high, low, none) and heating duration (long, short)—were varied, as shown in Supplementary Table S1, yielding 27 distinct soup products. Finally, 70–99 g of the tomato mix powder was added into 1 L of boiling water and afterward simmered for 5 min and occasionally stirred.

All soups were given a unique sample label to make sure the three platforms (i.e., sensory, GC-MS and LC-MS) all used the same identifiers.

After the preparation of the soups, sensory evaluation was done within two hours. In the same timeframe, sufficient subsamples were collected for both the GC-MS and LC-MS analysis, and a pooled sample (QC sample) of selected soups was prepared for both instrumental platforms. Further details of how the quality control (QC) samples were made are indicated in Supplementary Table S1. After sampling, aliquots of all individual and pooled samples were stored and shipped under frozen conditions until the instrumental analysis.

### 2.2. Sensory Analysis Description

Quantitative descriptive analysis (QDA) was used to investigate a total of 26 sensory attributes in the categories of odor, flavor, mouthfeel, aftertaste and afterfeel. The scoring of each attribute was recorded using EyeQuestion (logic8 Version 5.4.5, Build 2701) in the range from 0–100. Each of the 14 experienced panelists tested the 27 products in 4 separate sessions, for which all products were freshly prepared, and offered one-by-one to the panelist according to an incomplete, balanced design that was specifically developed to assess all products in each session. The samples were kept at 60 °C until testing and served in 50 mL plastic cups.

### 2.3. Liquid Chromatography–Mass Spectrometry

For the determination of non-volatile metabolite levels, an untargeted LC-MS method was used. To ensure the homogeneity of the tomato soup, each sample was vortexed before pipetting and the lower end of the pipette tip was cut off to prevent clogging. Tomato soup samples were prepared for LC-MS analysis by adding 900 µL methanol to 200 µL of tomato soup to precipitate the proteins. Samples were then mixed and spun down, and 900 µL of the supernatant was transferred and split into two equal aliquots. The samples were dried overnight at 43 °C in a SpeedVac. Dried extracts were reconstituted in 25 µL MilliQ (MQ) water (Milli-Q advantage A10, Merck, Rahway, NJ, USA) and 25 µL acetonitrile (Ultra LC-MS, Actuall Chemicals, Hoogeveen, The Netherlands) containing diclofenac, prednisolone and mycophenolic acid (SMerck Life Science NV, Amsterdam, The Netherlands) as non-endogenous internal standards (final concentrations were 0.125, 0.125 and 0.1 µg/mL, respectively). Some (12) samples were prepared in quadruplets for monitoring the reproducibility/precision for a total of 96 samples measured per batch.

After the preparation, the samples were randomized and run in 2 batches, which included QC samples, sample replicates and blanks. QC samples were analyzed every 10 samples and used to assess the data quality and monitor the instrument response.

Metabolites were separated using a Shimadzu Nexera UHPLC (Darmstadt, Germany) equipped with a Waters AccQ-Tag C18 column (100 mm, 2.1 µm). A 15 min gradient was applied as detailed in Supplementary Table S2, with mobile phase A consisting of MQ water + 0.1% formic acid and mobile phase B consisting of acetonitrile + 0.1% formic acid. The column was kept at a temperature of 60 °C, the autosampler temperature was kept at 12 °C and 1 µL samples were injected.

Mass spectrometry was conducted using a Sciex X500R QTOF mass spectrometer (Framingham, MA, USA) with ESI ionization in positive and negative modes. The QTOF was calibrated every 2 h during measurements. All measurements were performed with the following settings: curtain gas = 35, ion spray voltage = 5500 V (pos) and −4500 V (neg), temperature = 600 °C, ion source gas 1 = 60 psi, ion source gas 2 = 50 psi, collision energy = 5 V with a mass range from 100–1200 m/z.

Sciex .wiff2 raw data files were converted to mzml files using MSconvert (ProteoWizard toolkit, v.3.0) [16] and processed using XCMS (version 3.10) [17]. The resulting XCMS feature tables underwent manual inspection and further filtering, e.g., to remove isotopes and features occurring in blank samples. Details of the XCMS parameters and filtering are provided in Supplementary Table S3. The processed data were log-transformed, technical replicates were averaged and features were autoscaled prior to the multivariate analysis. For high-ranking sensory markers in selected models, LC-MS peak inspection was conducted using the Sciex OS software (version 1.7). This included confirmation of the chromatographic peak integration, careful inspection of each spectrum to extract the most probable formula, comparison of ionization modes and investigation of in-source fragmentation patterns. The identification confidence level of the annotated LC-MS features was based on the levels 1–5 proposed by Schymanski et al. (2014) [18]. Putative identification at levels 3–4 (formula-driven) was assessed in terms of the retention time plausibility and in the context of the chemical plausibility to be found in the matrix or produced by yeast. Level 1 identifications were validated by spiking a tomato soup sample with authentic standards and comparing the retention times and fragmentation patterns. The following databases were utilized during the compound investigation and annotation: Metlin [19], MoNA (mona.fiehnlab.ucdavis.edu, accessed on 1 June 2020), NIH PubChem [20], FooDB (www.foodb.ca, accessed on 1 June 2020), YMDB [21] and KNApSAcK [22].

### 2.4. Gas Chromatography–Mass Spectrometry

The composition of volatile compounds in the different tomato soups was determined by analyzing the headspace of the soups using solid-phase microextraction (SPME) followed by gas chromatography–mass spectrometry (GC-MS) analysis. Tomato soups were defrosted on a roller bench and then sonicated for 10 min to ensure homogenization. Several quality control samples (QCs), which were a mix of a few selected tomato soup types, were repeatedly analyzed along the sequence to test the performance of the analysis. The extraction with SPME and the GCMS analysis followed the same procedure as described by Diez-Simon et al. (2020) [23]. In brief, volatiles from the headspace of the different tomato soups were trapped on an SPME fiber (Polydimethylsiloxane/Divinylbenzene/Carboxen; 50/30 μm diameter, 1 cm length (Supelco, Bellefonte, PA, USA)), which was thermally desorbed in the CIS containing an empty glass liner (1 mm ID) with a helium flow of 1 mL/min at 280 °C for 2 min onto the GC column in splitless mode. The analyses were conducted on an Agilent GC7890A coupled to a 5975C quadrupole mass spectrometer. The column used was a Zebron ZB-5MSplus with dimensions 30 m × 0.25 mm × 1.00 µm (Phenomenex, Torrance, CA, USA). The column oven was temperature-programmed starting at 45 °C for 2 min, then increased at a rate of 5 °C/min to 250 °C and then maintained at 250 °C for 5 min. The column effluent was ionized via electron impact at 70 eV in the scan range m/z 33–500.

All 27 tomato soups were analyzed in a randomized order. An empty glass vial and a blank (water) sample were measured at the beginning of the series. Quality control samples (QCs) were repeatedly analyzed once every six samples. The raw GC-MS data were pre-processed using an untargeted metabolomics approach, which was also described in detail before [23]. In brief, raw data were baseline-corrected and mass peaks of samples were aligned using MetAlign software [24].

Signal redundancy was removed and mass spectra were reconstructed using MSClust [25]. 

Retention indices were calculated based on a series of n-alkanes (C_8_–C_22_) using a third-order polynomial function. Volatile compounds were identified by matching the mass spectra and RI to authentic reference standards or those in the NIST17 Mass Spectral library (v.2.3) following the criteria for metabolite identification proposed by Sumner et al. (2007) [26]. Compounds that did not fit the criteria were annotated as non-identified.

### 2.5. Sensory Data Processing

To correct for differences between assessors regarding scaling effects and offsets, the sensory data was standardized one sensory attribute at a time. Given a dataset with *I* assessors, *J* products and *K* sensory attributes, the intensity $y_{ijk}$ assessed for attribute *k* by panelist *i* for product *j* was standardized to ${\tilde{y}}_{ijk}$ as follows:

(1) $${\tilde{y}}_{ijk}=\frac{y_{ijk}-{\bar{y}}_{ik}}{s_{ik}}$$

Here, *ȳ_ik_* and *s_ik_* are the mean intensity and standard deviation for attribute *k* and panelist *I* across the products *J*, respectively [27]. For easier interpretability, the standardized intensities *ỹ_ijk_* were back-transformed to restore the original means and respective standard deviations:

(2) $${\tilde{y}}_{ijk}^{*}={\bar{y}}_{k}+{\tilde{y}}_{ijk}*sp_{k}$$

Here, $\bar{y}$*_k_* refers to the overall mean for attribute *k* and *sp_k_* refers to the pooled standard deviation for attribute *k* across the *I* assessors:

(3) $$sp_{k}=\sqrt{\frac{\sum_{i=1}^{I}s_{ik}}{I*\left(J-1\right)}}$$

To test the effects of standardization and evaluate the variation between products for regression, the F-value of the product effect was obtained using a mixed ANOVA in R 4.0 [28] by considering the product effect as a fixed variable and the assessor effect as a random variable. For further analyses, the product effects $\nu_{jk}$—subsequently termed sensory responses—were calculated as the mean across assessors for each product and attribute:

(4) $$v_{jk}=\frac{\sum_{i=1}^{I}{\tilde{y}}_{ijk}^{*}}{I}$$

### 2.6. Statistical Analysis

The multivariate data analysis was conducted using the MUVR package [29] in R 4.0. This package uses a repeated double cross-validation (rdCV) framework [30] to eliminate features iteratively and rank them by their importance and to define a minimal-optimal (“min”) and an all-relevant (“max”) set of predictors. The importance of the mass spectrometry features for modeling sensory responses and for discriminating between product compositions was estimated using partial least squares discriminant analysis (PLS-DA) for classification, partial least squares regression (PLS-R) for regression on the LC-MS data and random forests for regression on the GC-MS data. The LC-MS dataset contained multiple highly correlated features for single chemical compounds, whereas, for the GC-MS, the features were more condensed.

Features were ranked by their variable importance in projection (VIP) for the PLS models and by mean decrease in accuracy for the random forest regression. For each variation in composition and preparation, a PLS-DA model was generated to distinguish between the respective factor levels, minimizing the number of misclassifications in a 4-fold rdCV with 500 repetitions and assessing the final model performance using the balanced accuracy (*BACC*). The *BACC* for a classification problem with I levels was calculated using

(5) $$BACC=\frac{\sum_{i=1}^{L}TPR_{i}+TNR_{i}}{2L}$$

where *TPR_i_* and *TRN_i_* are the true positive and true negative rates, respectively, for the *i*th level. Each sensory attribute was modeled separately, minimizing the root-mean-squared error of prediction (RMSEP) in a 9-fold cross-validation with 200 repetitions, and the final model performance was assessed by calculating the *Q*² value. The *Q*² value was calculated using

(6) $$Q^{2}=1-\frac{PRESS}{TSS}=1-\frac{\sum_{j=1}^{J}{\left(y_{j}-{\hat{y}}_{j,-j}\right)}^{2}}{\sum_{j=1}^{J}{\left(y_{j}-\bar{y}\right)}^{2}}$$

where ${\hat{y}}_{j,-j}$ represents the prediction of the *j*th response by a model for which the *j*th sample was excluded during fitting.

## 3. Results

In this study, 27 different soup compositions were deliberately chosen to investigate the effects of each of the five compositional variables. These included different strengths of the soup base, as well as different dosages of yeast-derived flavor products. Corn and olive oil were used to study whether they would influence specific food attributes, and two different heating procedures were tested for their impact on sensory profiles. This study was designed to analyze a broad variety of sensory attributes, shown in Supplementary Figure S1, which illustrates the scoring range of each flavor attribute in all the soups with yeast-derived products. Certain sensory attributes were potentially more interesting than others when evaluating a soup basis, including garlic, onion, intensity and umami flavor. Based on the design of the soup varieties, it was anticipated that the developed models could help with the prediction of flavor.

### 3.1. Explorative Analysis

**Figure 2:**
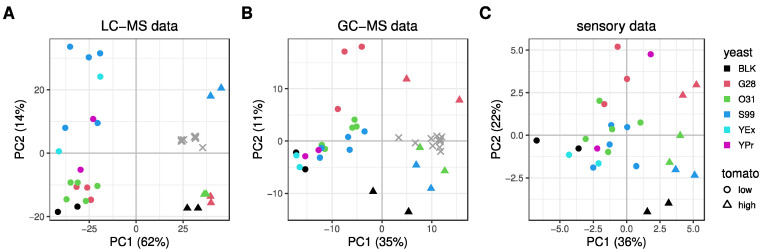
PCA score plots for all three platforms. Colors represent the added yeast-derived process flavors and shapes represent the tomato dosage. BLK: tomato soup without yeast-derived products. (**A**) Log-transformed, autoscaled LC-MS data. (**B**) Log-transformed, autoscaled GC-MS data. (**C**) Autoscaled sensory response.

To determine the driving factor behind the separation of the samples for the different data sets, PCA plots were made (Figure 2). An additional figure is included in the supplementary data that displays the PCAs of LC-MS and GC-MS, including the QC samples, demonstrating the repeatability of our platforms (Supplementary Figure S2). In the sensory analysis, as well as in the analysis of the volatile (GC-MS) and non-volatile and semi-volatile (LC-MS) metabolites, the soups containing a high tomato dosage were separated from those having a low dosage along PC1, while along PC2, the different yeast types could be partially distinguished (Figure 2). Other variables included in the study design had a lower impact on this PCA separation.

The LC-MS PCA (Figure 2A) shows the dramatic impact of the tomato dosage driving the separation, and soups with yeast S99 were also clearly different from other yeast-derived products. For the GC-MS data (Figure 2B), the primary source of separation was the tomato dosage and the secondary source was the type of yeast-derived product, especially G28. Likewise, in the PCA of sensory response data (Figure 2C), yeast G28 seemed most distinct from the other yeast products and samples were clearly separated by tomato dosage. The different grouping between LC-MS and GC-MS in the specific yeast-derived products showed the complementarity of our platforms.

### 3.2. Classification Performance of the Soup Compositions on All Platforms

**Figure 3:**
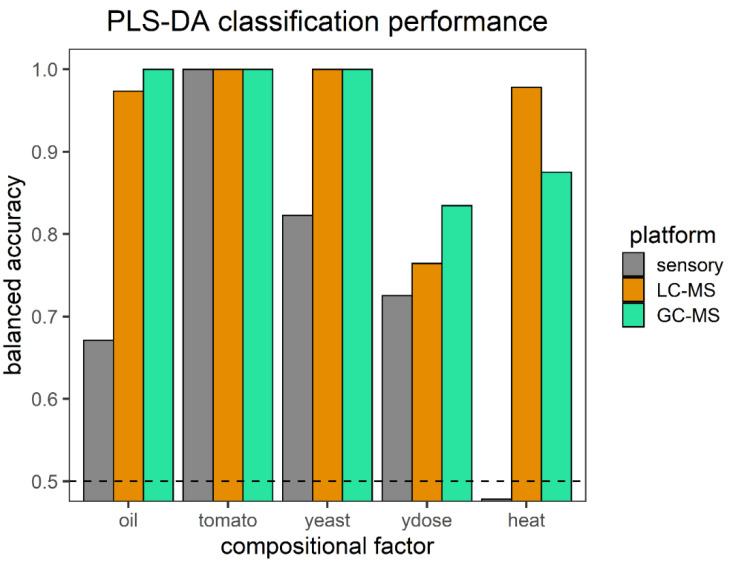
The three platforms used and their classification performance expressed as the balanced accuracy for each of the compositional factors of the soups to represent how well different compositional factors could be distinguished. Values below 0.5 can be considered random. Oil: oil type, tomato: tomato dosage, yeast: yeast-derived flavor product, ydose: yeast-derived flavor product dosage, heat: heating time.

To evaluate how changes in soup composition were reflected by the three platforms, PLS-DA classifiers were trained using the different datasets. Their performance in distinguishing the five compositional factors in our range of soups is presented in Figure 3. Classification performance was represented as balanced accuracy, with higher values indicating better performance, and for values below 0.5, it was not possible to use the platform to model this compositional factor. As to be expected from the PCA analyses, all three platforms distinguished very well between different levels of tomato content in the samples, which showed that this had a major impact on the outcome of our chemical and sensory measurements. GC-MS and LC-MS also readily distinguished the oil and type of yeast-derived product. LC-MS could differentiate better between heat duration treatment and GC-MS performed better on the dosage of yeast-derived products. Oil type and the yeast-derived product seemed to be difficult to distinguish using sensory measurements and heat seemed to have no influence on the sensory responses at all. Using the MS datasets, the dosage of the yeast product was the hardest to distinguish. The prediction of yeast dosage across different yeast types was challenging compared with the other compositional variables. This classification performance would be improved if there were MS features in common and around the same level between the yeast products but not present in other ingredients.

### 3.3. Discriminative Performance of the Sensory Panel

To evaluate how well the sensory attributes assessed by the sensory panel could differentiate between the 27 different soups, the F-ratio of the product effects for each sensory attribute was obtained by fitting a linear mixed model to the given quantitative descriptive analysis (QDA) data.

**Figure 4:**
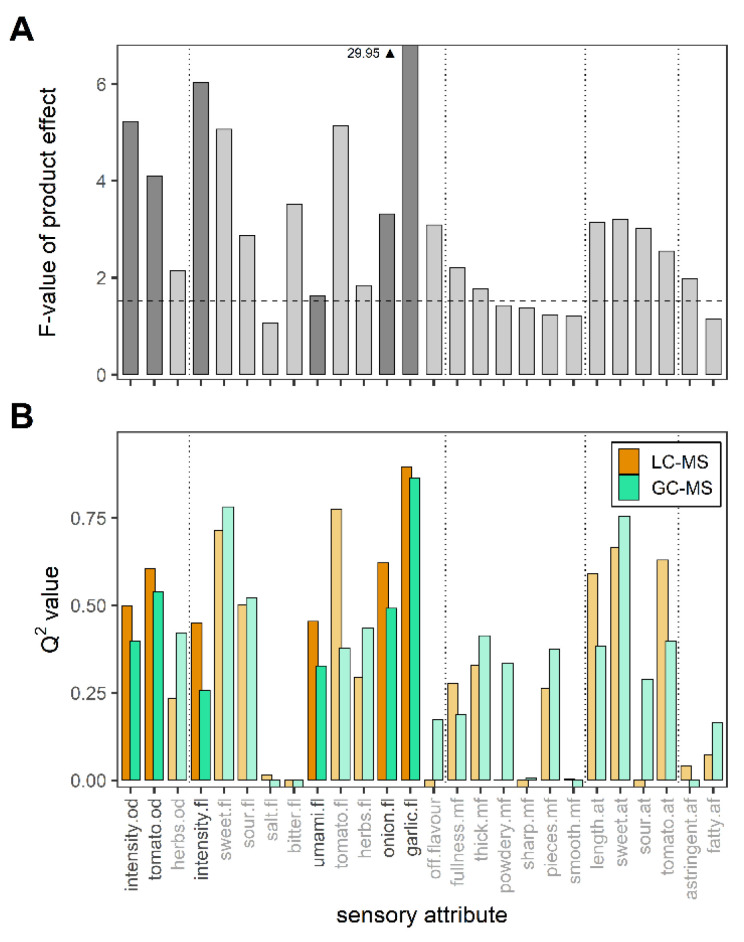
F-values of the product effect obtained from the sensory panel data using a mixed ANOVA model. (**A**) The horizontal dashed line represents the critical F-value for α = 0.05. Q^2^ values for the LC-MS and GC-MS predictive models are shown for each of the 26 sensory attributes. (**B**) Highlighted attributes were selected for further investigation. Abbreviations: _.af: afterfeel, _.at: aftertaste, _.mf: mouthfullness, _.fl: flavor, _.od: odor.

The F-values describe how well the soups could be distinguished according to different attributes, with higher F-values indicating that products could be distinguished more clearly. An F-value lower than the critical F-value essentially meant that no difference could be detected between products. The panelists could distinguish the products using certain sensory attributes better than others. As Figure 4A shows, the highest F-values were obtained for the attributes bitter flavor, sweet flavor, onion flavor, garlic flavor, tomato flavor and odor, and intensity flavor and odor. Garlic flavor scored exceptionally high and was most easily distinguished by the sensory panel.

### 3.4. Relationship between Metabolomics Platforms and Sensory Attributes

To quantify the model performance of LC-MS and GC-MS metabolite data in predicting sensory attributes, Q^2^ values were calculated. The higher the Q^2^ values, the better a sensory attribute could be predicted using a set of volatile or non-volatile metabolites detected in the tomato soups. The Q^2^ values of LC-MS and GC-MS models of each sensory attribute can be found in Figure 4B.

When the F-values of the sensory panel for all sensory attributes were compared to the Q² values, we found a clear relationship. High F-values for intensity odor; tomato odor and intensity; and tomato, onion and garlic flavors corresponded with high Q² values. If the taste panel could not distinguish well between the different soups for certain sensory attributes, it can be expected that the developed models of the correlated data sets would have low predictive value.

We made a selection of the sensory attributes for further investigation and annotation. For both LC-MS and GC-MS, onion flavor, garlic flavor and intensity flavor were selected for the strength of their models and umami flavor was added because it is a typical sensory attribute for yeast and tomato products. Additionally, intensity odor and tomato odor were included specifically for the GC-MS evaluations. Tomato flavor and sweet flavor were not further investigated here because tomato flavor is closely related to tomato odor and the LC-MS method was not designed for the relevant carbohydrates.

### 3.5. Annotation of Features in LC-MS and GC-MS

The LC-MS and GC-MS features in the models were ranked by their importance for the prediction of sensory attributes. For the selected attributes, the ranking values of features can be found in Supplementary Tables S5 and S6 and a heat map presenting the levels of high-ranking features in each soup composition can be seen in Figure S5. Lower rank values indicate higher importance in the model (and a high rank for that model).

The most important LC-MS features per sensory attribute were tentatively annotated using m/z and retention time (level 5 identification), spectral investigation using isotope and adduct information (level 4), and additional information based on chemical databases and MS library comparison to in-source fragmentation data (level 3) [17]. Table 1 presents the annotations of the highest-ranking features in each selected model. Further details of putative identifications are presented in Supplementary Table S4. GC-MS features were putatively annotated by comparison to the standard NIST library and Kovats indexes (see Supplementary Table S6). Table 2 provides a summary of the highest-ranking GC-MS features and their putative annotations.

Many of the high-ranking features of the onion flavor and garlic flavor model were putatively identified as γ-glutamyl conjugated peptides or peptide derivatives. Most of these were scientifically described as garlic-like or onion-like flavor compounds and are natural constituents of garlic and onion-family vegetables. The identification of these types of compounds in the tomato soups was not unexpected given that the products included onion (O31) and garlic (G28) yeast-derived flavors. Additionally, γ-glutamyl peptides, especially the sulfur-containing ones, are also strongly linked to the kokumi feeling [12]. Kokumi-related compounds can enhance flavor via a contribution to the mouthfeel, although not having taste themselves [31], contributing to food’s richness. They have been associated with a savory flavor and “meatiness”, enhancing umami and sweet flavors.

In the garlic flavor model, many features were highly abundant in the G28 samples, and annotations were dominated by sulfur-containing compounds in the LC-MS analysis and polysulfides in the GC-MS analysis. The top-ranking LC-MS feature for garlic flavor was confirmed (level 1 identification) using an authentic standard of methionyl-proline (Met-Pro) (Supplementary Figure S3). Interestingly, Met-Pro was previously reported to enhance salty taste [32]. 

The putative identifications of highly ranked features in the model of umami flavor included the products of non-enzymatic browning ribose-isoleucine (ranked second) and ribose-glycine (ranked seventh) [33]. Non-enzymatic browning usually happens during the storage or cooking of food and is associated with Maillard reactions. Zhou et al. (2021) confirmed that Maillard reaction intermediates were positively correlated with umami and salt taste [34]. 

In the intensity flavor model, the second-ranking LC-MS feature was identified (level 1, Supplementary Figure S4) as asparagine-leucine (Asp-Leu), which was reported to contribute to umami taste [35]. The top 5 most important GC-MS features of this sensory attribute included two aldehydes, including 2-methyl-2-butenal, which was highly ranked in the GC-MS model for intensity odor and reported to be found in food items like tomato, herbs and spices.

Other features that were generally highly ranked in the selected GC-MS sensory attributes included thiophenes, pyrazines and terpenes, which were also important contributors to flavor and aroma [36]. The GC-MS annotations of this study are described in more detail by Diez-Simon et al. (2020) [23]. 

It was interesting to see correlations between GC-MS and LC-MS features, which were especially pronounced for organosulfur compounds. For example, in the garlic flavor models, the fourth-ranked LC-MS feature, putatively identified as S-(allylthio)cysteine, correlated with the fifth-ranked GC-MS feature di-allyl trisulfide (Pearson R correlation = 0.974). Similarly, γ-glutamyl-S-allylthio-cysteine and γ-glutamyl-S-propylcysteine (ranked 6th and 13th, resp., in the LC-MS) correlated with the methyl allyl trisulfide (SPME6081, correlation factor R = 0.977 and R = 0.971 resp.) seen in the GC-MS analysis (although not significant for the garlic model). The feature γ-glutamyl-S-propylcysteine also correlated with methyl allyl disulfide (SPME2148, correlation factor R = 0.952) in the GC-MS analysis. These correlations increased the confidence in the biochemical relevance of the annotations, relating them to common precursors and metabolic pathways. For example, γ-glutamyl-S-alkyl-cysteine is a precursor of S-allylcysteine and diallyl polysulfides in garlic [37,38]. 

## 4. Discussion

This study was designed to investigate whether food metabolomics can be used to recognize and predict specific sensory attributes. If this is possible, metabolomics could (partially) replace the testing of new food products by taste panels and contribute to the development of new products with specific flavor characteristics.

Our study revealed that minor tomato soup recipe variations affected flavor perception and that this could not only be detected by the human sensory panels but could also be linked to changes in the chemical compositions as measured using metabolomics platforms. The LC-MS and GC-MS measurements of tomato soups could clearly distinguish the different soups and our models could show which changes were driving the classification performance. However, well-performing prediction models still rely on products that can be well-distinguished by trained sensory panels and the chemical features that are linked to the sensory attributes. This highlights the importance of a well-designed study that includes a sufficient diversity of food samples, together with clearly defined sensory attributes, a well-trained panel and appropriate coverage of the analytical platforms.

**Figure 5:**
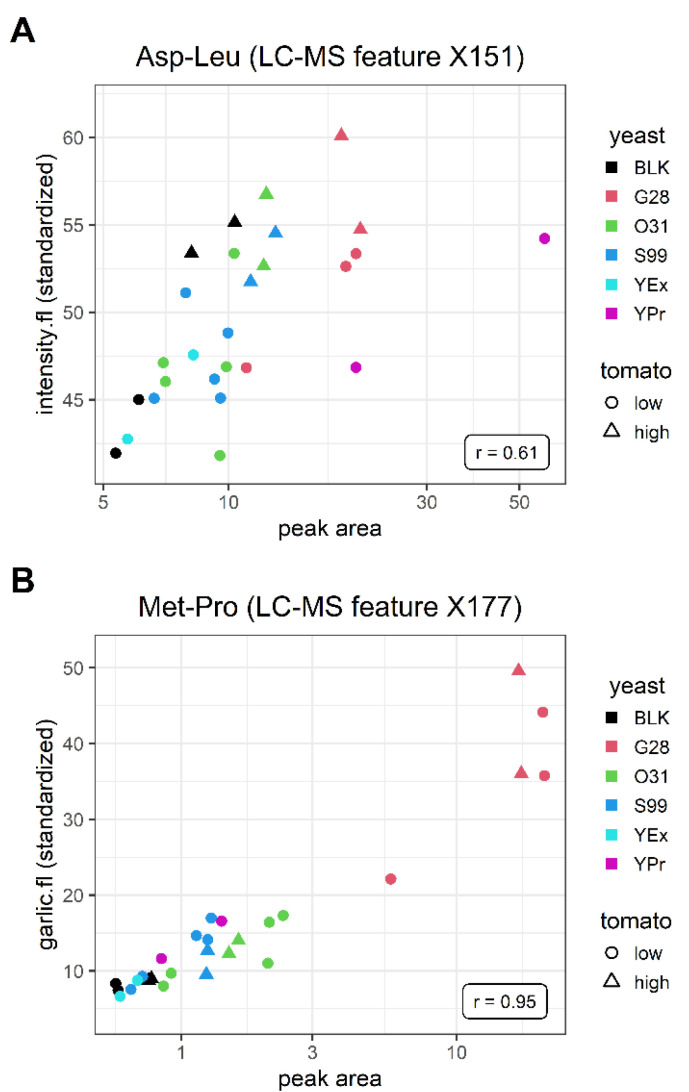
Standardized sensory scores plotted against the peak areas of the identified features Asp-Leu (X177) (**A**) and Met-Pro (X151) (**B**). Colors represent the added yeast extracts/process flavors and shapes represent tomato dosage. BLK: tomato soup without yeast-derived products. The R-value for Asp-Leu excluding the YPr outliers was 0.74.

Our models suggested several chemical features that were linked to the perception of specific flavor attributes. This can be illustrated with two dipeptide examples that showed a linear relationship between the standardized sensory scores and the log-transformed peak areas of the identified features (Figure 5). Although dipeptides are generally known to affect flavor perception, these specific molecules were not previously linked to the respective sensory attributes [39,40,41,42,43]. 

Such new relationships might allow for fine-tuning of food compositions to the desired flavor profile.

In conclusion, we showed the feasibility of combining sensory data, LC-MS and GC-MS platforms to find new information related to flavor perception in a complex food matrix. For the purpose of this study specifically, the obtained annotations and confirmation of correlations for Met-Pro with garlic flavor and Asp-Leu with intensity flavor demonstrated the applicability of our approach. We demonstrated the strength of untargeted metabolomics analysis that was utilized for biomarker discovery of specific sensory attributes. Future directions will include the validation of the sensory contribution of the annotated markers via food spiking experiments followed by sensory panel testing. In this manner, such a molecular-based approach can ultimately steer product development toward a preferred sensory profile, which can ultimately be evaluated by humans.

## Figures and Tables

**Table 1:** Putative identifications of the top 5 highest-ranked LC-MS features of the sensory attribute models selected for further investigation.

	Onion Flavor			Garlic Flavor			Umami Flavor			Intensity Flavor		
No.	Feature	Putative Identification ^1^	Id. Level ^2^	Feature	Putative Identification ^1^	Id. Level ^2^	Feature	Putative Identification ^1^	Id. Level ^2^	Feature	Putative Identification ^1^	Id. Level ^2^
1	X1313 X71	γ-Glu-Met ^3^	4	X177	Met-Pro	1 ^4^	X1414	5’-S-Acetyl-2’-deoxy-5’-thiouridine ^3^	4	X1163	PC(16:1/2:0) or an isomer	4
2	X1317	γ-Glu-Val	4	X141	N-monopropionyl-cystine	4	X1650	Ribose-isoleucine product	4	X151	Asp-Leu	1 ^4^
3	X1544 X280	γ-Glu-Leu or -Ile ^3^	3	X36	Isomer of S-allyl-Cys	3 ^5^	X153	N2,N2-Dimethylguanosine	4	X1787	Gln-Gln-His-His	4
4	X28	γ-Glu-aminopropiononitrile	4	X152	S-(allylthio)-Cys	4	X2033	Gln-Val-Lys-Glu-Leu	5	X2223	Feruloyltyramine	4
5	X216	methyl xanthine derivative	4	X1908 X663	Modified Ala-Ala peptide ^3^	4	X893	Ala-Ala-Pro-Val-Ala-Ala-Lys	5	X418	Tetrahydro-1-methyl-beta-carboline-3-carboxylic acid (MTCA)	4

No.: rank position in the top 5; Id. level: identification confidence level. ^1^ If the putative identification is an oligopeptide, different sequences of the peptides are also possible, except for those confirmed with a reference standard. ^2^ The identification confidence levels of the annotated LC-MS features were based on the levels 1–5 proposed by Schymanski et al. (2014) [17]. ^3^ This feature was represented twice in the top 10 of highest-ranking features, once measured in positive and once in negative ionization mode. ^4^ Comparison of the MS/MS spectrum with that of the reference standard confirmed the identity, but the exact isomer could not be confirmed. ^5^ Comparison of the MS/MS spectrum with that of the reference standard of S-allyl-cysteine disconfirmed that to be the identity of this feature.

**Table 2:** Putative identifications of the top 5 highest-ranked GC-MS features of the sensory attribute models selected for further investigation.

	Onion Flavor			Garlic Flavor			Umami Flavor		
No.	Feature	Putative Identification/Annotated Formula	Id. Level ^1^	Feature	Putative Identification/Annotated Formula	Id. Level ^1^	Feature	Putative Identification/Annotated Formula	Id. Level ^1^
1	SPME362	3-Methylthiophene	2	SPME5189	C_8_H_12_N_2_	3	SPME3741	3-Carene	2
2	SPME3540	Trimethylpyrazine	2	SPME2617	C_6_H_16_N_2_	3	SPME8427	Δ-Elemene	2
3	SPME2617	C_6_H_16_N_2_	3	SPME6357	C_8_H_10_N_2_O	3	SPME8624	α-Cubebene	2
4	SPME4906	C_8_H_8_O_2_	3	SPME6926	C_6_H_8_S_2_	4	SPME4062	D-Limonene	1
5	SPME2106	C_4_H_6_O	4	SPME8203	Di-allyl-trisulfide	2	SPME4134	β-Phellandrene	2
	**Intensity Flavor**			**Intensity Odor**			**Tomato Odor**		
**No.**	**Feature**	**Putative Identification/Annotated Formula**	**Id. Level ^1^**	**Feature**	**Putative Identification/Annotated Formula**	**Id. Level ^1^**	**Feature**	**Putative Identification/Annotated Formula**	**Id. Level ^1^**
1	SPME8112	C_13_H_28_	4	SPME8112	3-Methylthiophene	2	SPME5189	C_8_H_12_N_2_	3
2	SPME7856	α-Ethylidene-benzeneacetaldehyde	2	SPME7856	C_4_H_6_O	4	SPME1092	C_7_H_12_O	3
3	SPME4788	4-Methyl-benzaldehyde,	2	SPME4788	C_6_H_16_N_2_	3	SPME7223	C_5_H_7_BrO	4
4	SPME5834	C_11_H_20_	4	SPME5834	2-Methyl-2-butenal	2	SPME7181	C_3_H_4_S_3_	4
5	SPME3115	C_8_H_14_O_2_	3	SPME3115	Dimethyl-disulfide	1	SPME4823	3-Ethyl-2,5-dimethylpyrazine	2

No.: rank position in the top 5; Id. level: identification confidence level. ^1^ The identification confidence levels of the annotated GC-MS features were based on the levels 1–4 proposed by Sumner et al. [25].

## Data Availability

The original raw data is provided at www.ebi.ac.uk/metabolights/MTBLS6068.

## Supplementary Materials

The following supporting information can be downloaded at: https://www.mdpi.com/article/10.3390/metabo12121194/s1. Figure S1: Original sensory scores of all samples and flavor attributes; Figure S2: Quality control of LC-MS and GC-MS data using PCAs; Figure S3: MS/MS spectra of Asp-Leu in tomato soup 05–1 mirrored against a pure standard; Figure S4: MS/MS spectra of Met-Pro in tomato soup 19–2 mirrored against a pure standard; Figure S5: LC-MS metabolomic features appearing in all soup variations; Supplementary Table S1: Ingredient characteristics of the tomato soups used in this study; Supplementary Table S2: Gradient conditions of the LC-MS method; Supplementary Table S3: XCMS parameter settings and feature filtering; Supplementary Table S4: LC-MS annotations of the most significant features in the models for onion flavor, garlic flavor, umami flavor and intensity flavor; Supplementary Table S5: LC-MS features and their ranking values for selected sensory attributes; Supplementary Table S6: GC-MS features and their ranking values for selected sensory attributes.
