# Supplementary material for: Flavor Profiling Using Comprehensive Mass Spectrometry Analysis of Metabolites in Tomato Soups
Source: Metabolites. 2022 Nov 29;12(12):1194. doi: 10.3390/metabo12121194 (PMC9788410; doi:10.3390/metabo12121194)
Supplement: Supplementary file 1 [file metabolites-12-01194-s001.zip › Supporting Information S1.pdf]

Supporting Information

Flavor profiling by comprehensive mass spectrometry analysis of metabolites in tomato soups

Simon Leygeber[1], Justus L. Grossmann[2], Carmen Diez-Simon[3], Naama Karu[1], Anne-Charlotte Dubbelman[1], Amy C. Harms[1], Johan A. Westerhuis[2], Doris M. Jacobs[4], Peter W. Lindenburg[1,5], Margriet M.W.B. Hendriks[6], Brenda Ammerlaan[6], Marco A. van den Berg[7], Rudi v Doorn[7], Roland Mumm[1,8], Robert D. Hall[1,3,8], Age K. Smilde[2], Thomas Hankemeier[1]*

[1]Leiden Academic Centre for Drug Research, Leiden University, Einsteinweg 55, 2333 CC Leiden, The Netherlands

[2]Swammerdam Institute for Life Sciences, University of Amsterdam, Science Park 904, 1098 XH, Amsterdam, The Netherlands

[3]Laboratory of Plant Physiology, Wageningen University and Research, Droevendaalsesteeg 1, Wageningen, 6708 PB, The Netherlands

[4]Unilever Foods Innovation Centre, Bronland 14, Wageningen, 6708 WH, The Netherlands

[5]Leiden Center for Applied Bioscience, University of Applied Sciences Leiden, Zernikedreef 11, 2333 CK Leiden, The Netherland

[6]DSM Biodata & Translation, Alexander Fleminglaan 1, Delft 2613 AX, The Netherlands

[7]DSM Food & Beverages, Alexander Fleminglaan 1, Delft 2613 AX, The Netherlands

[8]Wageningen Research (Bioscience), Wageningen University and Research, Droevendaalsesteeg 1, The Netherlands

*Thomas Hankemeier, hankemeier@lacdr.leidenuniv.nl

List of figures

Figure S1 Original sensory scores of all samples and flavor attributes

Figure S2 Quality control of LC-MS, GC-MS data by PCAs

Figure S3 MS/MS spectra of Asp-Leu in tomato soup 05-1 mirrored against a pure standard

Figure S4 MS/MS spectra of Met-Pro in tomato soup 19-2 mirrored against a pure standard

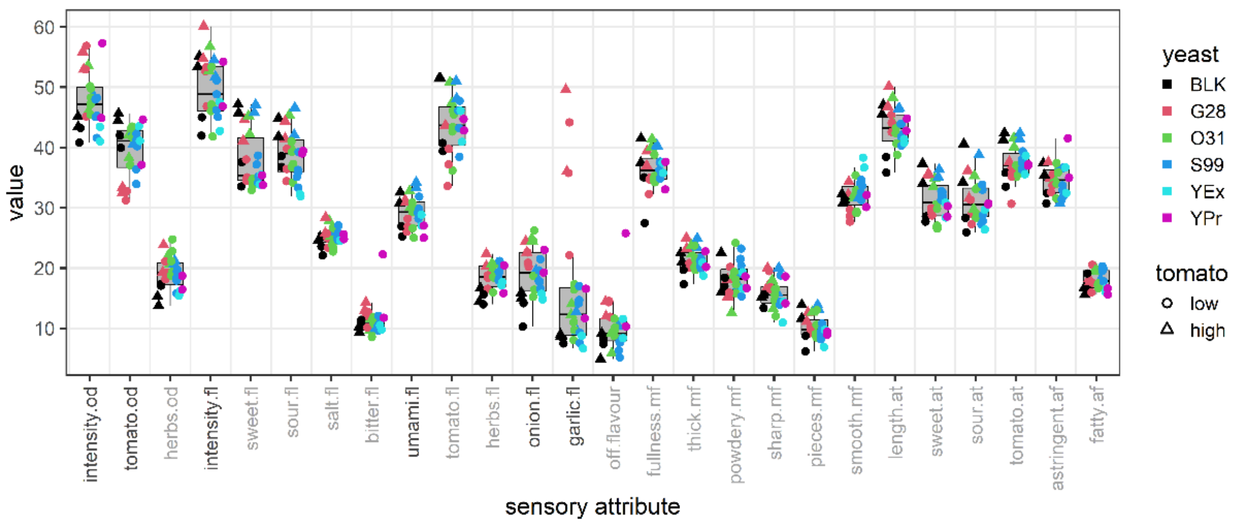

Figure S1: Original sensory scores of all samples and flavor attributes. Colors represent the added yeast derived process flavors and shapes represent tomato dosage. BLK= tomato soup without yeast derived products.

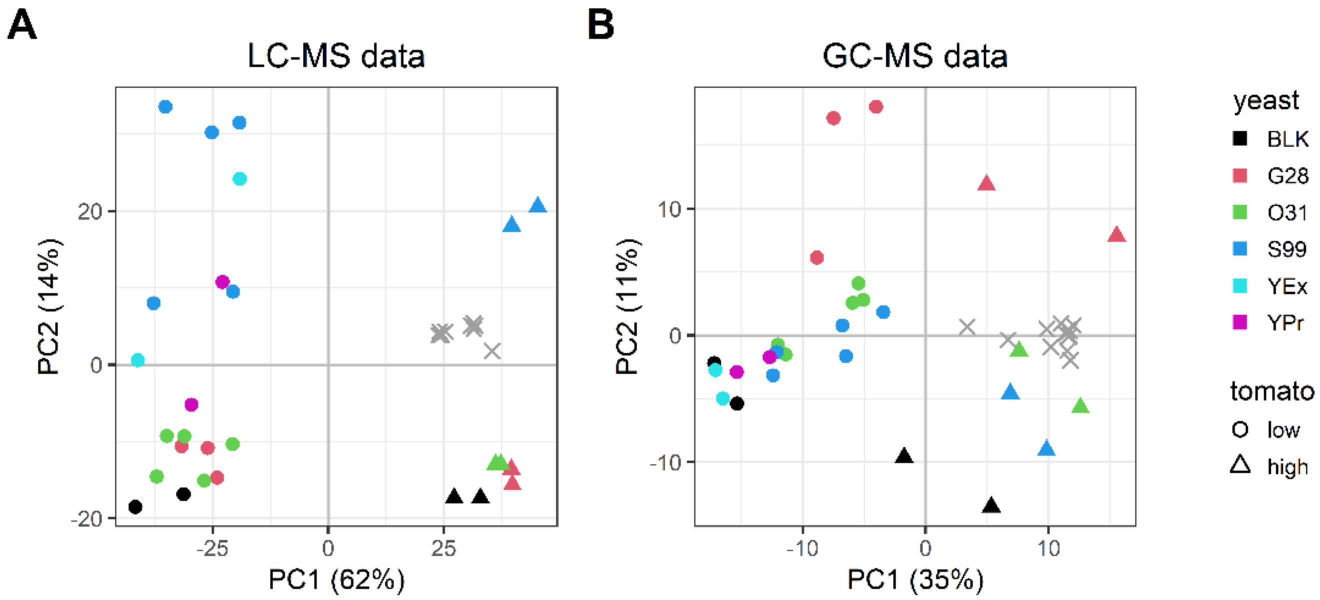

Figure S2: PCA score plots including the quality control samples (QCsamples) as grey crosses which were produced as indicated in table S1 . Colors represent the added yeast derived process flavors and shapes represent tomato dosage. BLK= tomato soup without yeast derived products. A: Log-transformed, autoscaled LC-MS data. B: Log-transformed, autoscaled GC-MS data.

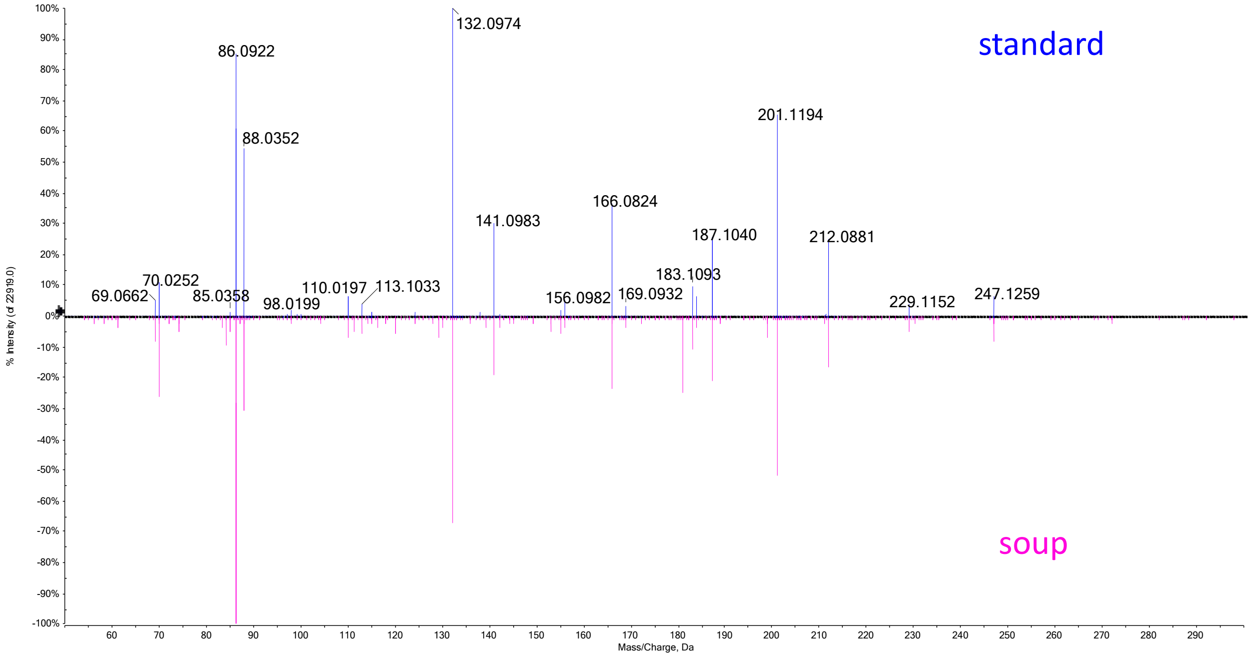

Figure S3 MS/MS spectra of Asp-Leu in tomato soup 05-1 mirrored against a pure standard

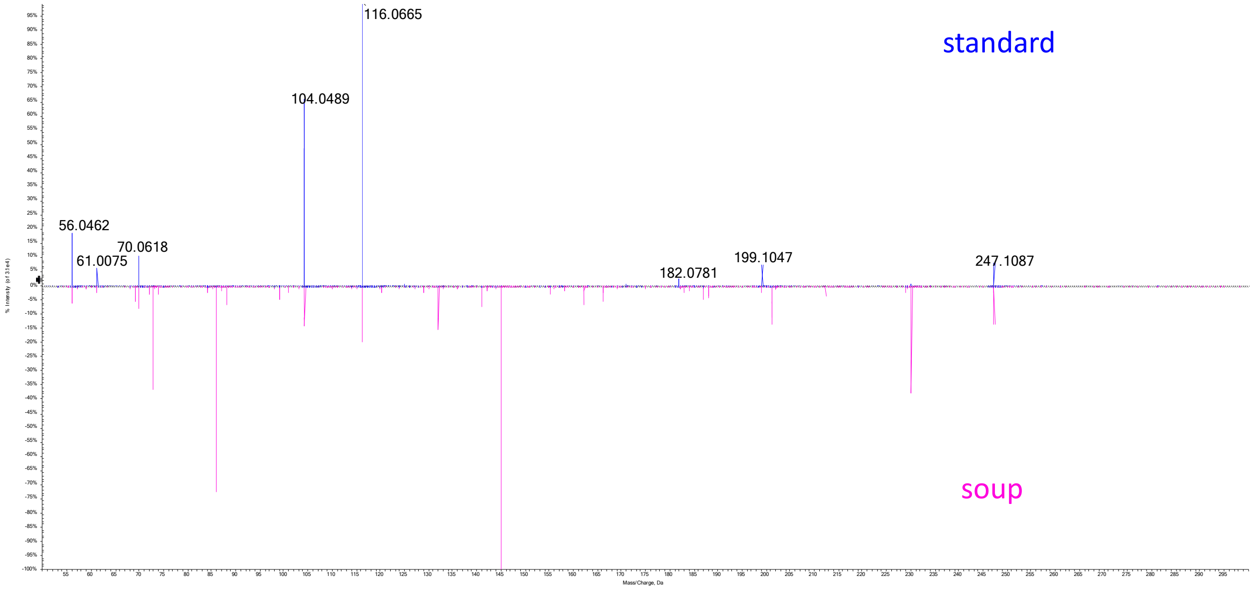

Figure S4 MS/MS spectra of Met-Pro in tomato soup 19-2 mirrored against a pure standard
